# Annual acknowledgement of manuscript reviewers

**DOI:** 10.1186/s13012-015-0253-x

**Published:** 2015-06-10

**Authors:** Anne Sales, Michel Wensing

**Affiliations:** Implementation Science, London, UK

## Abstract

The editors of *Implementation Science* would like to thank all our reviewers and editors who have contributed to the journal in Volume 9 (2014).

**Reviewers**

**Eivind Aakhus**

Norway

**Michele Abendstern**

United Kingdom

**Purva Abhyankar**

United Kingdom

**Sarah Alderson**

United Kingdom

**Lisa Arai**

United Kingdom

**Stephanie Archer**

United Kingdom

**Rebecca Armstrong**

Australia

**Melissa Armstrong**

United States of America

**David Aron**

United States of America

**Kelly Aschbrenner**

United States of America

**Thanos Athanasiou**

United Kingdom

**Lou Atkins**

United Kingdom

**Ross Bailie**

Australia

**David Baker**

United States of America

**Luciana Ballini**

Italy

**Melanie Barwick**

Canada

**Mark Bayley**

Canada

**Steven Belenko**

United States of America

**Derek Bell**

United Kingdom

**Sara Bennett**

United States of America

**Guillermo Bernal**

United States of America

**Joanne Beswick**

United States of America

**Fiona Beyer**

United Kingdom

**Christian Blickem**

United Kingdom

**Gary Bond**

United States of America

**Debbie Bonetti**

United Kingdom

**Andrew Booth**

United Kingdom

**Marije Bosch**

Australia

**Xavier Bosch-Capblanch**

Switzerland

**Peter Bower**

United Kingdom

**Jennifer Boyko**

Canada

**Daniel Bradford**

United States of America

**Jeffrey Braithwaite**

Australia

**Paula Brauer**

Canada

**Jamie Brehaut**

Canada

**Carinne Brody**

United States of America

**Helen Brooks**

United Kingdom

**C Hendricks Brown**

United States of America

**Angela Buchholz**

Germany

**Helen Burchett**

United Kingdom

**Jako Burgers**

Netherlands

**André Bussières**

Canada

**Phyllis Butow**

Australia

**Bernard Candas**

Canada

**Martin Cartwright**

United Kingdom

**Trudie Chalder**

United Kingdom

**Catherine Chamberlain**

Australia

**Duncan Chambers**

United Kingdom

**John Chatwin**

United Kingdom

**Elaine Chen**

United States of America

**Sudeh Cheraghi-Sohi**

United Kingdom

**Timothy R. Church**

United States of America

**David Clarke**

United Kingdom

**Evelyn Cornelissen**

Canada

**Angela Coulter**

United Kingdom

**Geoffrey Curran**

United States of America

**Janet Curran**

Canada

**Laura Damschroder**

United States of America

**Teresa Damush**

United States of America

**Marina Davoli**

Italy

**Wout de Boer**

Switzerland

**Sarah Dennis**

Australia

**Marjolein Dieleman**

Netherlands

**Vance Dietz**

United States of America

**Don Dillman**

United States of America

**Irina Dinca**

Sweden

**Francois Dionne**

Canada

**Carmen D Dirksen**

Netherlands

**Diane Dixon**

United Kingdom

**Mamuka Djibuti**

Georgia

**Peggy Dolcini**

United States of America

**Linying Dong**

Canada

**Shannon Dorsey**

United States of America

**Dawn Dowding**

United States of America

**Judith Dyson**

United Kingdom

**Carolyn Ehrlich**

Australia

**Moriah Ellen**

Israel

**Carole A. Estabrooks**

Canada

**Matthew Exline**

United States of America

**Tracey Farragher**

United Kingdom

**Greg Fell**

United Kingdom

**Rashida Ferrand**

United Kingdom

**Caroline Finch**

Australia

**Patricia Fontaine**

United States of America

**Jane Forman**

United States of America

**Yvonne Forsell**

Sweden

**Christina Foss**

Norway

**Claire Foster**

United Kingdom

**Robbie Foy**

United Kingdom

**Gerdien Franx**

Netherlands

**Simon French**

Canada

**John Gabbay**

United Kingdom

**Anna Gagliardi**

Canada

**Benjamin Gardner**

United Kingdom

**Val Gebski**

Australia

**Charles Glisson**

United States of America

**Christina Godfrey**

Canada

**Gaston Godin**

Canada

**Rachel Gold**

United States of America

**Ralph Gonzales**

United States of America

**Lynne Goodacre**

United Kingdom

**Sophie Goyet**

Cambodia

**Ian Graham**

Canada

**Eric Granholm**

United States of America

**Carla A Green**

United States of America

**Trisha Greenhalgh**

United Kingdom

**Karin Guldbrandsson**

Sweden

**Martin Hagger**

Australia

**Jacqueline Halladay**

United States of America

**Stephen Halloran**

United Kingdom

**Alison Hamilton**

United States of America

**Nik Sherina Hanafi**

Malaysia

**Margaret Handley**

United States of America

**Steven Hanna**

Canada

**Claire Harris**

Australia

**Jocelyn Harris**

Canada

**Mark Harris**

Australia

**Jo Hart**

United Kingdom

**Gill Harvey**

United Kingdom

**Lewis Hassell**

United States of America

**Henna Hasson**

Sweden

**Masahiro Hayakawa**

Japan

**Emma Healey**

United Kingdom

**James Heiby**

United States of America

**Susanne Hempel**

United States of America

**Jane Hendy**

United Kingdom

**Benjamin Henwood**

United States of America

**Reece Hinchcliff**

Australia

**Tammy Hoffmann**

Australia

**Bev J Holmes**

Canada

**Susan Huckson**

Australia

**Carmel Hughes**

United Kingdom

**Michael Hurlburt**

United States of America

**Dominic Hurst**

United Kingdom

**Alison Hutchinson**

Australia

**Sylvia Hysong**

United States of America

**Alfonso Iorio**

Canada and Italy

**Wanrudee Isaranuwatchai**

Canada

**George Jackson**

United States of America

**Roberta James**

United Kingdom

**Lianne Jeffs**

Canada

**Patrick Jeurissen**

Netherlands

**Marie Johnston**

United Kingdom

**Caroline Jones**

United Kingdom

**Susanne Kaae**

Denmark

**Eleftherios Kaklamanos**

Greece

**Monika Kastner**

Canada

**Anne Kennedy**

United Kingdom

**Bridie Kent**

United Kingdom

**Stefan Kertesz**

United States of America

**Amy Kilbourne**

United States of America

**Jan Koetsenruijter**

Netherlands

**Niina Kolehmainen**

United Kingdom

**Anita Kothari**

Canada

**Robert Krell**

United States of America

**Peter Lachman**

United Kingdom

**Sue Latter**

United Kingdom

**Julie Leask**

Australia

**JuHee Lee**

Korea, South

**Martin Lee**

United States of America

**Natalie Leon**

South Africa

**Li Li**

United States of America

**Zhanming Liang**

Australia

**Chuan-Fen Liu**

United States of America

**John Lloyd**

France

**Craig Lockwood**

Australia

**Fabiana Lorencatto**

United Kingdom

**Julie Lowery**

United States of America

**Graeme Maguire**

Australia

**Donna Mak**

Australia

**Graham Martin**

United Kingdom

**Steve Martino**

United States of America

**Carl May**

United Kingdom

**Patrick Mbindyo**

Kenya

**Sara McCafferty**

United Kingdom

**Annie McCluskey**

Australia

**Eleanor McConnell**

United States of America

**Mark McGovern**

United States of America

**Amy McPherson**

Canada

**Theodorus (Dirk) Mettes**

Netherlands

**Sandy Middleton**

Australia

**Rhona Mijumbi**

Uganda

**Anne Miles**

United Kingdom

**Robin Miller**

United Kingdom

**Brian Mittman**

United States of America

**Kaelan Moat**

Canada

**Julia Moore**

Canada

**Andrew Morden**

United Kingdom

**Elizabeth Murray**

United Kingdom

**Lakshmi Murthy**

United Kingdom

**Janet Myers**

United States of America

**Juliet Nabyonga-Orem**

Uganda

**Chirk Jenn Ng**

Malaysia

**Laura Nichols**

United States of America

**Per Nilsen**

Sweden

**Paul Norman**

United Kingdom

**Wynne Norton**

United States of America

**Katia Noyes**

United States of America

**Eivor Oborn**

United Kingdom

**Kathryn Oliver**

United Kingdom

**Newton Opiyo**

Kenya

**Lois Orton**

United Kingdom

**Andy Oxman**

Norway

**Nancy Padian**

United States of America

**Lawrence Palinkas**

United States of America

**Samuel Pannick**

United Kingdom

**Justin Parkhurst**

United Kingdom

**Neesha Patel**

United Kingdom

**Sanjay Patel**

United Kingdom

**Jeremy Pearson**

United Kingdom

**Robert Penfold**

United States of America

**Tahna Pettman**

Australia

**Susan Pickard**

United Kingdom

**David Pilgrim**

United Kingdom

**Brigitte Piso**

Austria

**Lourdes Planas**

United States of America

**Marie Polley**

United Kingdom

**Stephanie Polus**

Germany

**Maria Carmen Portillo**

Spain

**Edward Post**

United States of America

**Michael Potter**

United States of America

**Alison Powell**

United Kingdom

**Emma Power**

Australia

**Justin Presseau**

United Kingdom

**Carel Pretorius**

United States of America

**Enola Proctor**

United States of America

**Lisa Puchalski Ritchie**

Canada

**Andrew Quanbeck**

United States of America

**Craig Ramsay**

United Kingdom

**Nicole Rankin**

Australia

**Geetha Ranmuthugala**

Australia

**Tim Rapley**

United Kingdom

**Joanna Reynolds**

United Kingdom

**Alison Ritter**

Australia

**Nicola Robinson**

United Kingdom

**Louise Rohrbach**

United States of America

**Jo Rycroft-Malone**

United Kingdom

**Sanjeeve Sabharwal**

United Kingdom

**Mary Ann Scheirer**

United States of America

**Kate Seers**

United Kingdom

**Paul Shekelle**

United States of America

**Camille Short**

Australia

**Julius Sim**

United Kingdom

**Marko Simunovic**

Canada

**Ted Skolarus**

United States of America

**Karen Smith**

United Kingdom

**Samuel Smith**

United Kingdom

**Leif Solberg**

United States of America

**Sanjeev Sridharan**

Canada

**Liz Steed**

United Kingdom

**Ian Steen**

United Kingdom

**Israel Steiner**

Israel

**Mathilde Strating**

Netherlands

**Sharon Straus**

Canada

**Rachel Tabak**

United States of America

**Huibert Tange**

Netherlands

**Marcel Tanner**

Switzerland

**Faye Taxman**

United States of America

**Cath Taylor**

United Kingdom

**Natalie Taylor**

Australia

**Oleg Tcheremissine**

United States of America

**Gregory Teague**

United States of America

**Sally Theobald**

United Kingdom

**Siwan Thomas-Gibson**

United Kingdom

**Carl Thompson**

United Kingdom

**Graham Thornicroft**

United Kingdom

**Nhan Tran**

Switzerland

**Lyndal Trevena**

Australia

**Samson Tse**

Hong Kong

**Nikki Turner**

New Zealand

**Sami Uka**

Serbia

**Robin Urquhart**

Canada

**Thomas Valente**

United States of America

**Per Olav Vandvik**

Norway

**Patrick Verschueren**

Belgium

**Eleana Villanueva**

Peru

**Terri Voepel-Lewis**

United States of America

**Joshua Vogel**

Switzerland

**Sallie J. Weaver**

United States of America

**Thomas Webb**

United Kingdom

**Rosie Webster**

United Kingdom

**Bryan Weiner**

United States of America

**Elizabeth West**

United Kingdom

**Rene Westhovens**

Belgium

**Michael Wilson**

Canada

**Christopher Wise**

United States of America

**Miranda Wolpert**

United Kingdom

**Linda Worrall**

Australia

**Janet Yamada**

Canada

**Patsy Yates**

Australia

**Jennifer Yost**

Canada

**Ian Young**

United Kingdom

**Ken Zakariasen**

United States of America

**Pauline Zardo**

Australia

**Editors**

**Gregory Aarons**

United States of America

**Robbie Foy**

United Kingdom

**Signe Flottorp**

Norway

**Bridie Kent**

United Kingdom

**Susan Michie**

United Kingdom

**Denise O'Connor**

Australia

**Anne Rogers**

United Kingdom

**Nick Sevdalis**

United Kingdom

**Sharon Straus**

Canada

**Paul Wilson**

United Kingdom

